# Supplementary material for: SinGAN-Seg: Synthetic training data generation for medical image segmentation
Source: PLoS One. 2022 May 2;17(5):e0267976. doi: 10.1371/journal.pone.0267976 (PMC9060378; doi:10.1371/journal.pone.0267976)
Supplement: Attachment — Submitted filename: Response to Reviewers.pdf [file pone.0267976.s001.pdf]

Dear editor and reviewers,

Thank you very much for your valuable comments and time spent reviewing our manuscript. Please find our point-by-point responses below.

Response to the academic editor:

The paper needs significant improvement. It should position the proposed method with respect to the state of the art in GAN-based synthetic data generation - detail the contribution, show the advantages, put the results in comparison. It should also explain the improvement of results due to style transfer. More metrics should be employed to evaluate the generated images.

Answer:
We have addressed all these points based on the reviewers comments. The contribution is emphasised and new metrics like SIFID are introduced in the paper. More details for each change are discussed under the specific reviewer comments. We use a single image to train our GAN model. Therefore, we emphasised the superiority of our model which needs only a single image to train, over traditional GANs which usually require a large datasets to train. In this regard, we have skipped comparison with traditional GANs in this study. We are thankful for the comments and think that addressing them improved our manuscript significantly.

Reviewer 1:

Comment 1: First of all, the figures need to be significantly improved. Right now they are of very poor quality and very blurred. It is hard for the reviewers to make sense of the figures as they are. Use previously published PLOS ONE papers as a reference.

Answer: First we would like to thank you for your effort to review our paper. We have noticed the point you raised here. However, we have uploaded high quality images to the system separately. We assume that when the system generates the review version of the paper, some conversion has happened to the images. We hope that high quality images will appear in the camera ready version.

Comment 2: There are many spelling mistakes. Please run the manuscript through a spell check.

Answer: All authors of this manuscript have gone through the paper again to correct spelling mistakes and grammatical errors.

Comment 3: The proposed approach uses state-of-the-art generation approach SinGAN to generate synthetic images along with the corresponding ground-truth followed by a style transfer. This is not a significant novelty other than the part of generating the corresponding ground-truth.

Answer: Thank you for your comment regarding the novelty. As you mentioned, our main contribution is generating synthetic polyp images with the corresponding ground truth to tackle the costly and the time consuming data annotation problem. Our approach is

specifically useful when preparing ground truth for image segmentation would be difficult. Therefore, we show that our approach helps to prepare large synthetic datasets with the corresponding groundtruth compared to generating only images.

Comment 4: The last 2 contributions of the paper are not new:

a. "We show that synthetic images and corresponding mask images can improve the segmentation performance when the size of a training dataset is limited."
b. We show that synthetic data can achieve a very close performance to the real data when the real segmentation datasets are large enough."

Answer: We removed these two points as you suggested and highlighted only the main contributions.

Comment 5: Recent works such as [1 - 3] have already shown this with medical images. The references are missing.

[1] Theagarajan, R. and Bhanu, B., 2019. DeephESC 2.0: Deep generative multi adversarial networks for improving the classification of hESC. PloS one, 14(3), p.e0212849.

[2] Witmer, A. and Bhanu, B., 2018, October. HESCNET: A Synthetically Pre-Trained Convolutional Neural Network for Human Embryonic Stem Cell Colony Classification. In 2018 25th IEEE International Conference on Image Processing (ICIP) (pp. 2441-2445). IEEE.

[3] Jonnalagedda, P., Weinberg, B., Allen, J., Min, T.L., Bhanu, S. and Bhanu, B., 2021, January. SAGE: Sequential Attribute Generator for Analyzing Glioblastomas using Limited Dataset. In 2020 25th International Conference on Pattern Recognition (ICPR) (pp. 4941-4948). IEEE.

Answer: We have included the specific references in the revised version.

Comment 6: Quantitative evaluation of the generated synthetic images are required. The authors need to provide scores such as the FID and SIFID scores as used in the SinGAN paper.

Answer: Thank you for suggesting additional metrics. Based on the suggestion we added SIFID values for our synthetic dataset as discussed in the original SinGAN paper. We rely only on the SIFID because it is more relevant for GANs that use single images to train. New SIFID values are provided in Table 1 for pure output and after applying style transfer.

Comment 7: The results provided in Tables 1 and 2 are not sufficient. Since training data is very important, how are the qualities of the generated images verified in Tables 1 and 2? One suggestion would be to compute the quality metric (FID, SIFID, etc.) of the generated images and take only the top X% of the synthetic images for training.

Answer: We agree with your point. Therefore, we have calculated SIFID which is more relevant in this study as we use the modified SinGAN, and added a new table to the manuscript. This really helped us to improve the quality of our study. According to the SIFID values from different synthetic data sets, we noted that almost all the synthetic data after applying style transfer show very similar results. Therefore, selecting top images based on SIFID values is ignored for the rest of the experiments in the paper. We refer to the new Table 1 for more details.

Reviewer 2:

The authors have developed a method for generating synthetic data for medical image segmentation based on sinGAN and style transfer. It is an interesting study to deal with privacy and small dataset of medical images. However, there is no comparison with existing methods, and the superiority of the proposed method is not clear. The reviewer suggests the authors to revise the manuscript according to the following comments.

Answer: Thank you very much for your valuable comments and time spent to review our paper.

Commnet 1: SinGAN-Seg as data sharing technique. Please add a comparison of the proposed method with other data augmentation methods (e.g., conventional GAN). What is the advantage of the proposed method over the conventional GAN based synthetic data generation?

Answer: We have added a clear description of how our SinGAN-Seg which uses a single image to train, differs from other conventional GAN methods which use large datasets to train. We did not compare with other conventional GAN methods because SinGAN-Seg uses only one image to train the models and thus comparison with other methods that use more than one image would not be insightful. Therefore, we added an explanation about why SinGAN-Seg is an important model in the medical domain which has a limited amount of data in the discussion section.

Comment 2: SinGAN-Seg with small datasets. As mentioned above, please highlight the superiority of the proposed method compared to other common data augmentation methods for small datasets analysis, too.

Answer: Additional details and discussions are added in subsection “SinGAN-Seg with small datasets” in the discussion section.

Comment 3: Did you apply style transfer in the results of Table 2 and Fig.11? Please clarify how style transfer improves the results.

Answer: We have introduced a new table with SIFID value comparisons between with and without using the style transfer algorithm (See Table 1). We have used this new table and other segmentation results to clarify why style transfer is important in this work. Additional details were added for descriptions of Table 3 (old Table 2) and Fig. 11.

– End of the response to reviewers letter –
