# Supplementary material for: SinGAN-Seg: Synthetic training data generation for medical image segmentation
Source: PLoS One. 2022 May 2;17(5):e0267976. doi: 10.1371/journal.pone.0267976 (PMC9060378; doi:10.1371/journal.pone.0267976)
Supplement: Attachment — Submitted filename: Response to Reviewers.pdf [file pone.0267976.s002.pdf]

Dear editor and reviewers,

Thank you very much for your valuable comments and the time to review our manuscript again. Please find our point-by-point responses below.

Response to the academic editor:
Improvement is still needed regarding experimental comparison and formatting.

Answer: We have performed additional experiments and compared our method with other state-of-the-art GAN methods in a new section in the manuscript. All the results are included in the manuscript, and we think that it significantly improved our manuscript. Additional formatting changes were done to improve the manuscript.

Reviewer #1:
Thank you very much for taking the time to review our manuscript a second time and for adding your comments to improve our manuscript further.

1) The authors mentioned that they have uploaded better quality images, but I still do not see any improvement in the quality of the images.
Answer: We are sorry for any inconvenience caused by this. We uploaded the images as separate files into the system, where the system automatically adds them into the reviewer's copy. We also noted the low-quality images when checking the reviewers' copies, but we had no option to change them. Therefore, we hope that the camera-ready version will have better quality images. If you have access to the individual images, you can check them to see the real quality of the images.

2) There are still some minor spelling mistakes, please check one more time. Line 93 date -> data.
Answer: We went through the whole paper again and corrected many of spelling and grammar mistakes.

3) Table 1 in the revised manuscript is very poorly formatted. I cannot see anything, except the first column. Please check the manuscript before uploading for review!
Answer: We use latex to compile our manuscript, and to generate track changes, we have used Latexdiff to add track changes to the manuscript. We also noted that table formats are broken from this tool. However, we have uploaded our manuscript without any track changes as they guided us. This manuscript copy (without any track changes) has all the tables in proper order and proper formats. We can see the same issue in this submission as well. We have used the correct table in both tex files to overcome this issue. Then, we cannot see track changes in tables, but we have made some minor changes to the old tables to correct formatting issues. The completely new tables are presented with track changes.

4) The authors mention and discuss the quality of the generated images using SinGan-seg throughout the paper, but there is no comparison with other GAN related works. Hence it is not possible to validate if the proposed approach is significantly better in terms of quality, other than the fact that it requires only 1 training image.

Answer: We have performed additional experiments to generate synthetic polyp images and the corresponding masks using current state-of-the-art GAN models, namely DCGAN, Progressive GAN, and FastGAN. The new results, which include a figure to do visual comparisons of the new GANs and SinGAN, two tables comparing FID values calculated with the full dataset and small datasets, and one graph which shows the effect of the number of training data used with GANs are discussed in the revised manuscript. These additional materials improve the quality of our manuscript substantially. Thank you very much for directing us to add this useful comparison to the paper and improving the contribution.

4.1) Although SinGan-seg takes only 1 input to train compared to conventional GANs that require more images, the authors need to generate results using at least 2 - 3 other approaches that use conventional GANs (even if it requires more training images compared to SinGan-seg). We need to see if there is significant difference in quality of generated images between the different approaches. This comparison will greatly benefit the readers to understand if the difference in no. of training images Vs. quality of generated images is significantly different.
Answer: See answer to comment 4

4.2) Similarly compare the SIFID scores of SinGan-seg with these other approaches.
Answer: We have compared SinGAN with other GAN models using FID instead of SIFID because SIFID is not compatible with the other GANs, which generates random samples instead of one-to-one mapping in SinGAN-Seg.
